# Hindlimb unloading, a physiological model of microgravity, modifies the murine bone marrow IgM repertoire in a similar manner as aging but less strongly

**DOI:** 10.1186/s12979-023-00393-1

**Published:** 2023-11-20

**Authors:** Coralie Fonte, Pauline Jacob, Anne Vanet, Stéphanie Ghislin, Jean-Pol Frippiat

**Affiliations:** 1 Stress Immunity Pathogens Laboratory, UR 7300 SIMPA, Faculty of Medicine, Lorraine University, Vandoeuvre-lès, Nancy, France; 2 Université Paris Cité, CNRS, Institut Jacques Monod, F-75013 Paris, France

**Keywords:** Antibody, Spaceflight, Humoral immunity, Stress, Immune system, B lymphopoiesis, V(D)J recombination, Aging

## Abstract

**Background:**

The spaceflight environment is an extreme environment that affects the immune system of approximately 50% of astronauts. With planned long-duration missions, such as the deployment of the Lunar Gateway and possible interplanetary missions, it is mandatory to determine how all components of the immune system are affected, which will allow the establishment of countermeasures to preserve astronaut health. However, despite being an important component of the immune system, antibody-mediated humoral immunity has rarely been investigated in the context of the effects of the space environment. It has previously been demonstrated that 30 days aboard the BION-M1 satellite and 21 days of hindlimb unloading (HU), a model classically used to mimic the effects of microgravity, decrease murine B lymphopoiesis. Furthermore, modifications in B lymphopoiesis reported in young mice subjected to 21 days of HU were shown to be similar to those observed in aged mice (18–22 months). Since the primary antibody repertoire composed of IgM is created by V(D) J recombination during B lymphopoiesis, the objective of this study was to assess the degree of similarity between changes in the bone marrow IgM repertoire and in the V(D)J recombination process in 2.5-month-old mice subjected to 21 days of HU and aged (18 months) mice.

**Results:**

We found that in 21 days, HU induced changes in the IgM repertoire that were approximately 3-fold less than those in aged mice, which is a rapid effect. Bone remodeling and epigenetics likely mediate these changes. Indeed, we previously demonstrated a significant decrease in tibial morphometric parameters from day 6 of HU and a progressive reduction in these parameters until day 21 of HU, and it has been shown that age and microgravity induce epigenetic changes.

**Conclusion:**

These data reveal novel immune changes that are akin to advanced aging and underline the importance of studying the effects of spaceflight on antibody-mediated humoral immunity.

**Supplementary Information:**

The online version contains supplementary material available at 10.1186/s12979-023-00393-1.

## Background

Spaceflight-induced immune system dysregulation, coupled with limited clinical care, represents a clinical risk to crewmembers in future deep-space missions. Indeed, a study of medical data collected from 46 astronauts who spent six months on the International Space Station (ISS) showed that 46% of them encountered immunological problems such as hypersensitivities and infections [1].

However, despite being an important component of the immune system, antibody-mediated humoral immunity has rarely been investigated in the context of the effects of the space environment. Some studies have suggested that humoral immunity might be affected during a space mission, even though spaceflight and a ground-based analog of spaceflight (6° head-down tilt bed rest) did not have a major impact on human B-cell homeostasis [2, 3]. Indeed, it was recently shown that the IgM repertoire of two out of five analyzed cosmonauts was significantly modified during and after a prolonged mission on the ISS [4]. Another recent study suggested a trend toward reduced murine antibody binding site diversity as a result of spaceflight [5]. Other studies revealed that hindlimb unloading (HU), a physiological model of microgravity [6], affects the murine antibody repertoire after vaccination [7, 8]. Similarly, it was shown that a 5-month spaceflight affects amphibian antibody production in response to antigen stimulation [9–11] and reduces the frequency of somatic hypermutation that diversifies antibody binding sites to improve their affinity for the antigen [12].

The creation of a diverse antibody repertoire, which is essential for effective host protection, requires the V(D)J recombination process that takes place during B lymphopoiesis in adult bone marrow. V(D)J recombination associates antibody gene segments to create antibody genes. For example, to create a heavy-chain gene, it associates a variable (IGHV), a diversity (IGHD) and a joining (IGHJ) gene segment. This recombination is mediated by several effectors. The recombination-activating gene 1 and 2 (RAG1/2) proteins interact with recombination signal sequences (RSSs) situated in the 3′ region of IGHV, on both sides of IGHD and in the 5′ region of IGHJ segments and cause DNA breaks between coding IGHV, IGHD or IGHJ segments and the noncoding RSS, leading to the formation of hairpin-sealed coding ends [13]. Then, the Artemis protein complex opens hairpin loops, eventually creating palindromic sequences corresponding to short inverted repeats of the segment terminal sequence [14], and terminal deoxynucleotidyl transferase (TdT) adds nontemplated nucleotides to coding ends. This is followed by hybridization of single-stranded DNA on a few nucleotides, removal of overhangs, and ligation. The end product is the third complementarity-determining region (CDR3) of heavy chains, which is a highly variable region harboring many of the antigen binding sites.

Interestingly, it was demonstrated using larvae of the amphibian *P. waltl* that developed on the ISS [15], mice subjected to 21 days of HU [16], and mice that spent 30 days aboard the BION-M1 satellite [17] that B lymphopoiesis is decreased in real and simulated microgravity. Furthermore, Lescale et al. [16] showed that 21 days of HU led to a decrease in murine B lymphopoiesis that was markedly similar to that observed in aged (18–22 months) mice. These authors showed that, as in aged mice, HU induced a decrease in pro-B and pre-B cells. Furthermore, they found that this decrease was associated with impaired IL-7 signaling in murine pro-B cells, as previously reported in aged mice [18].

Given that the assembly of antibody heavy-chain gene segments by V(D)J recombination occurs at the pro-B stage [19] and that IL-7 signaling profoundly influences IGHV segment selection during V-to-DJ recombination [20], we wondered whether HU would affect the murine bone marrow IgM repertoire and V(D)J recombination in the same manner as aging. The objective of this study was therefore to assess the degree of similarity between changes in the bone marrow IgM repertoire and in the V(D)J recombination process in HU and aged mice. Our results reveal that 21 days of HU and aging altered the bone marrow IgM repertoire in a similar manner, but the effect of aging was stronger. They also show that changes in the use of antibody gene segments in mice subjected to 21 days of HU could not be attributed to a change in the V(D)J recombination process or to a stress response.

## Results

### Evaluation of stress in aged and HU mice

**Fig. 1:**
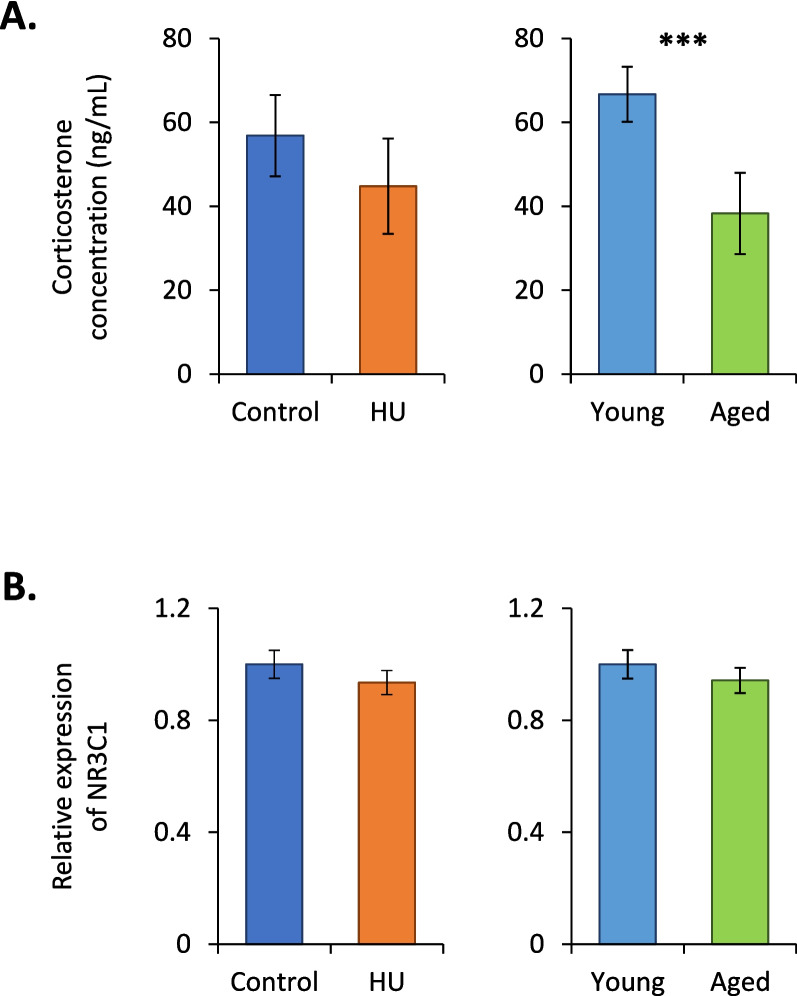
Evaluation of stress in HU and aged mice. **A** Corticosterone concentrations in sera, determined by ELISA, in control vs. HU (*n* = 10 in each group) and young vs. aged mice (*n* = 19 in each group). **B** Quantification, by quantitative real-time PCR, of NR3C1 mRNAs in the bone marrow of control vs. HU (*n* = 24 in each group) and young vs. aged mice (*n* = 27 in each group). This transcript encodes the glucocorticoid receptor. mRNA levels were normalized to those of 3 housekeeping transcripts. The relative value obtained with controls or young mice was set to 1. Data are shown as the means ± SDs. Statistically significant differences were found using Mann–Whitney or unpaired t tests. ***p ≤ 0.001

To evaluate the stress response, we quantified serum corticosterone, the major stress hormone in rodents, and glucocorticoid receptor (NR3C1) transcripts in the bone marrow because circulating and locally produced glucocorticoids can impact lymphopoiesis [21]. No difference in corticosterone concentration between control and HU mice was observed (Fig. 1A), as previously reported after 3 weeks of HU [16, 22]. There was a significant decrease in corticosterone concentration in aged mice, but the corticosterone concentrations remained close to those observed in unstressed mice (approximately 50 ng/mL, according to the literature). Figure 1B shows that there was no significant change in the relative expression of NR3C1 mRNAs in HU and aged mice. Taken together, these results suggest that our four groups of mice were nonsignificantly stressed, if at all.

### Impact of aging and HU on pro-B and pre-B cells

**Fig. 2:**
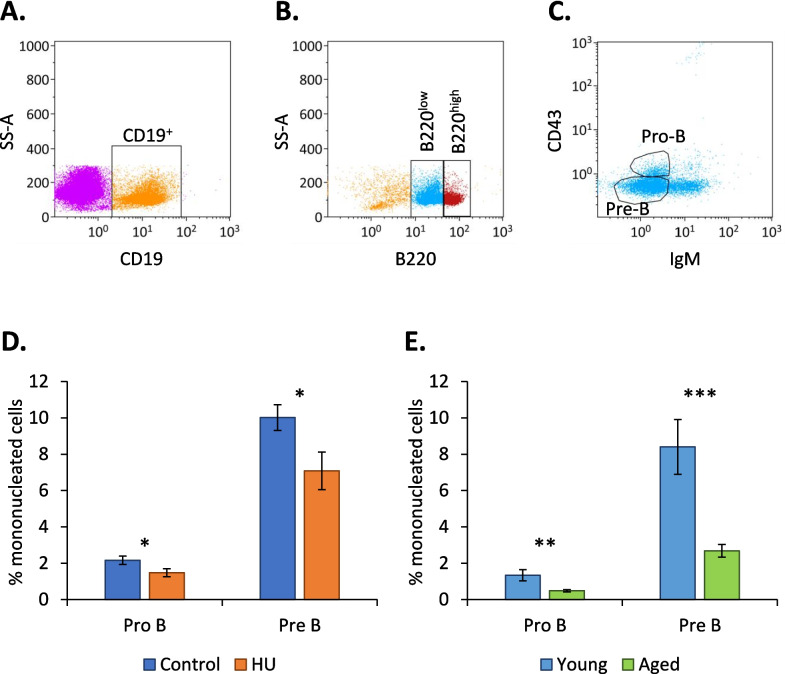
Quantification of pro-B and pre-B cells in the bone marrow of HU and aged mice. **A**,** B**,** C** Gates designed to identify pro-B and pre-B cells. These cells were identified based on the expression of the following markers: pro-B (CD19+/B220low/CD43+/IgM-) and pre-B (CD19+/B220low/CD43−/IgM-). **A** Selection of CD19+ lymphocytes from viable bone marrow cells. **B** Among CD19+ lymphocytes, cells could be divided into B220 high and B220 low populations. **C** In the B220 low population, depending on the expression of CD43 and IgM, the pro-B (CD43+/IgM-) and pre-B (CD43-/IgM-) populations could be distinguished. **D**, **E** Frequencies of pro-B and pre-B cells in the bone marrow of control (*n* = 14) vs. HU (*n* = 13) (**D**) and young (*n* = 13) vs. aged (*n* = 13) (**E**) mice. Data are shown as the means ± SDs. Statistically significant differences were found using Mann–Whitney or unpaired t tests. *p ≤ 0.05; **p ≤ 0.01; ***p ≤ 0.001

The impact of aging and HU on pro-B and pre-B cells was determined by flow cytometry. Significant decreases in both subpopulations were noted in both groups (Fig. 2). These results are consistent with the findings of the study by Lescale et al. [16], thereby validating our HU procedure and confirming that this treatment induces a decrease in the abundance of pro-B and pre-B cells. We also noted that pro-B and pre-B cells were more strongly affected by aging than by HU.

### Effects of aging and HU on the repertoire of IgM heavy chains

To investigate how HU and aging affect the IgM repertoire, we constructed libraries of IgM heavy-chain cDNAs from the bone marrow of HU and aged mice and from the bone marrow of corresponding 3.5-month-old controls (Control and Young groups). These libraries were subjected to high-throughput sequencing. Only functional sequences with unique CDR3s, the most diverse part of Ig transcripts, were retained to eliminate potential PCR biases and clonal expansions. We obtained 10,406 to 27,241 unique sequences per sample, of which 16–18% were nonfunctional. These nonfunctional sequences were not further considered, as the purpose of this study was to determine the impact of aging and HU on the expressed IgM repertoire. We also assessed the efficiency of the strategy used to create our libraries by determining the number of functional IGHV gene segments in our datasets. The International Immunogenetics Information System (IMGT) predicts 125 functional IGHV gene segments in the murine immunoglobulin heavy chain locus (*IGH*) located on chromosome 12 [23]. Additional file 1 shows that 130 functional IGHV gene segments were detected in our datasets. We are therefore confident that our libraries provide an accurate picture of IGH rearrangements. This is supported by previous studies that have shown that detected IGHV gene segments assessed without amplification parallel the repertoires reported using more focused amplification methods [24, 25].

**Fig. 3:**
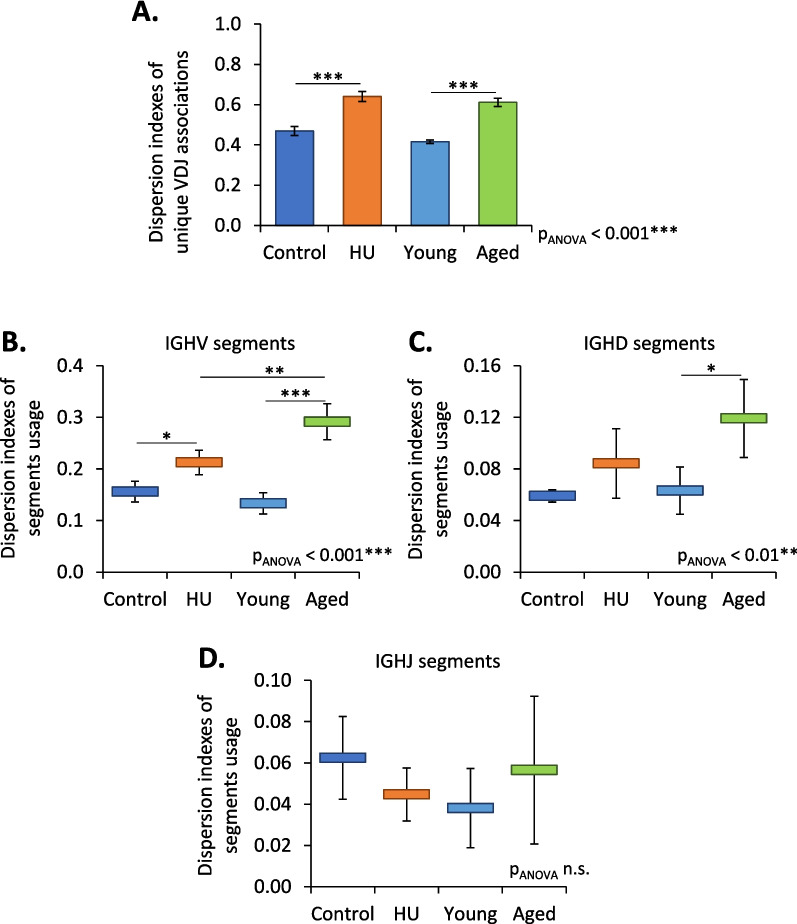
The IgM repertoire of HU and aged mice was different from that of the corresponding 3.5-month-old controls. **A** Dispersion indexes for unique VDJ associations in the bone marrow of control vs. HU and young vs. aged mice. **B**, **C**, **D** Dispersion indexes for IGHV (**B**), IGHD (**C**) and IGHJ (**D**) segment usage in the bone marrow of control vs. HU mice and young vs. aged mice. Data are shown as the means ± SDs of 4 groups, each comprising 5 mice (*N* = 4, *n* = 5). Statistically significant differences were found using one-way ANOVA followed by a Tukey–Kramer post hoc test. *p ≤ 0.05; **p ≤ 0.01; ***p ≤ 0.001; n.s., nonsignificant

To determine how aging and HU affect the IgM repertoire, dispersion indexes (DIs) were calculated. These indexes vary from 0 (no diversity) to 1 (greatest diversity). Because the two independent HU experiments and the study of aged mice could not be performed simultaneously, several 3.5-month-old control groups had to be used. Thus, we first checked the stability of the bone marrow IgM heavy chain repertoire in these control groups. The lack of significant difference in DIs for the use of IGHV, IGHJ, and IGHD segments (Additional file 2), the similarity in CDR3 lengths (Additional file 3A), and the similar amino acid composition of CDR3s of 11 to 14 aa (Additional file 3B), except the 13 aa CDR3s for which a small statistically significant difference in DI of 0.02 ± 0.01 (mean ± SD) was noted, indicates the stability of the bone marrow IgM heavy-chain repertoire in the 3.5-month-old C57BL/6 J control groups. Then, we compared DIs for unique VDJ associations in the bone marrow of control vs. HU and young vs. aged mice. Figure 3A shows that the IgM repertoire of HU and aged mice was different from that of the corresponding 3.5-month-old controls. We also studied the usage of IGHV, IGHD and IGHJ segments. Figure 3B shows that the use of IGHV segments was modified in HU and aged mice, but with greater modification in aged mice (difference in DI of 0.06 ± 0.024 (mean ± SD) in HU mice and difference in DI of 0.16 ± 0.04 in aged mice versus the corresponding 3.5-month-old controls), while the use of IGHD segments (Fig. 3C) was modified only in aged mice, and no statistically significant modification of IGHJ segment usage was found in either group (Fig. 3D). Taken together, these data show that aging and HU modify the IgM repertoire in the bone marrow, that these modifications are mainly due to changes in the use of IGHV segments and that, within 21 days, HU induces changes in the IgM repertoire that are approximately 3-fold less than those in aged mice, which is a rapid effect.

### IGHV segment usage according to their position in the murine *IGH* locus

**Fig. 4:**
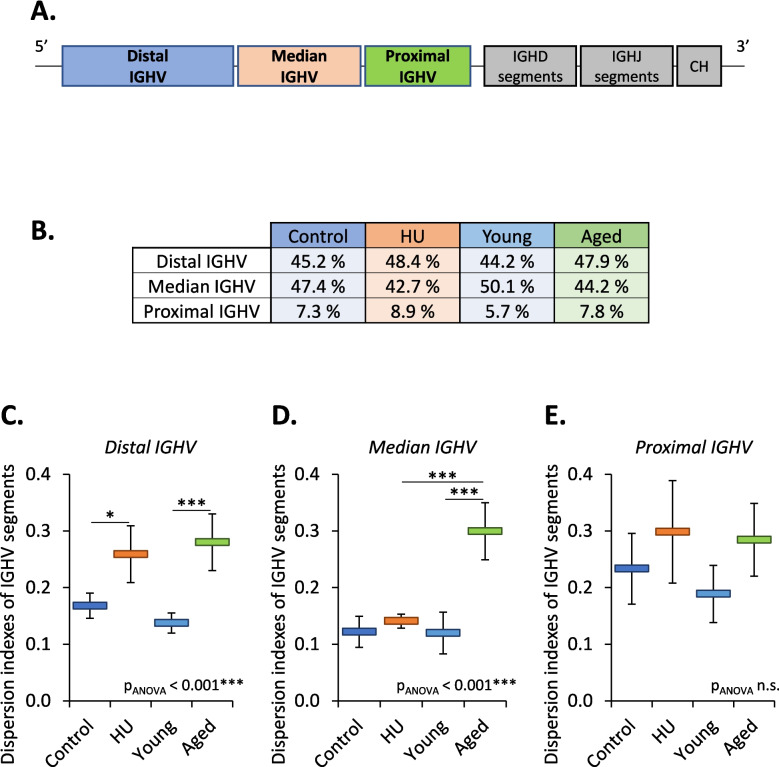
IGVH segment usage according to their location on the murine *IGH* locus. **A** Schematic presentation of distal, median and proximal IGHV segments on the murine *IGH* locus. **B** Percentage of IGHV segments belonging to the distal, median and proximal areas in our four groups of mice. **C**, **D**, **E** Dispersion indexes for distal (**C**), median (**D**), and proximal (**E**) IGHV segment usage in control vs. HU and young vs. aged mice. Data are shown as the means ± SDs of 4 groups, each comprising 5 mice (*N* = 4, *n* = 5). Statistically significant differences were found using one-way ANOVA followed by a Tukey–Kramer post hoc test. *p ≤ 0.05; ***p ≤ 0.001; n.s., nonsignificant

An important parameter in the choice of IGHV segments during V(D)J recombination is the location of the segment on the locus, which is subdivided into 3 zones (from 5′ to 3′: distal, median, and proximal) (Fig. 4A) [26, 27]. Of the 130 functional IGHV segments detected in our libraries, 62% belonged to the distal zone, 26% belonged to the median zone, and 12% were part of the proximal zone. These distal, middle and proximal functional IGHV segments were present in 44–48%, 43–50% and 6–9% of the unique VDJ associations found in our libraries, respectively (Fig. 4B). This observation confirms a bias in the use of IGHV segments during V(D)J recombination. Consequently, we investigated the effects of aging and HU on IGHV segment usage based on the zones that these segments belonged to. Figure 4C shows that the use of distal IGHV segments was modified in HU and aged mice. Modification of median IGHV segment usage was noted in aged but not in HU mice (Fig. 4D), while there was no difference in proximal IGHV usage (Fig. 4E). These data suggest that the changes in IGHV segment usage in HU mice depicted in Fig. 3B mainly resulted from changes in the use of distal IGHV segments, while changes in IGHV segment usage in aged mice were due to changes in the use of IGHV segments belonging to the distal and median zones of the murine *IGH* locus.

### Potential impact on immunity

**Fig. 5:**
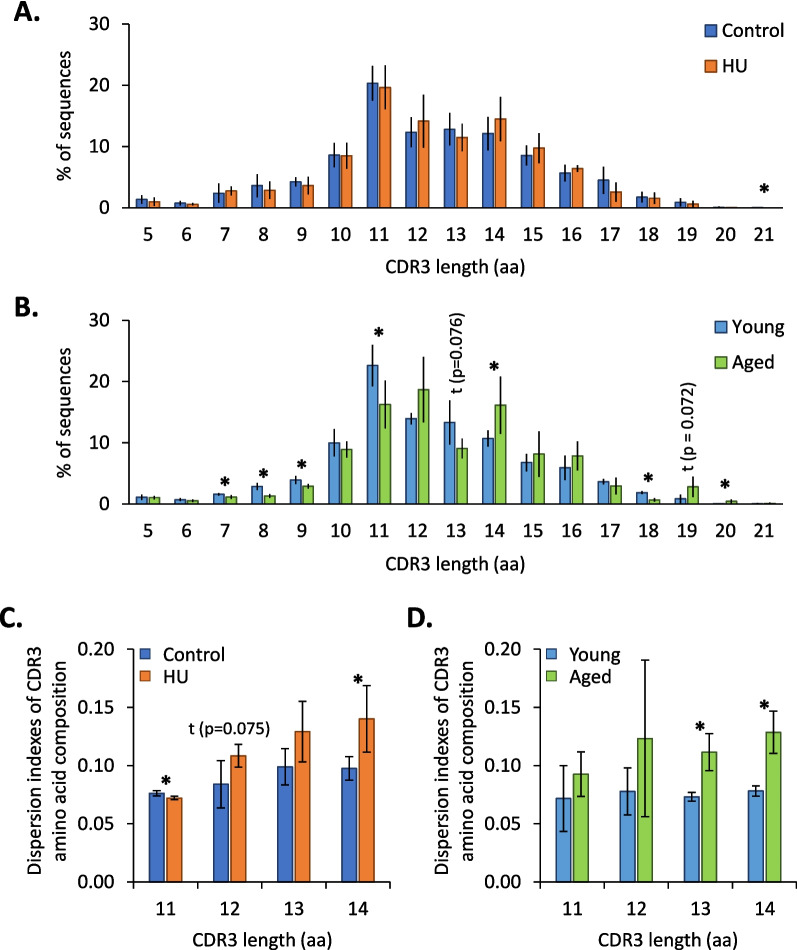
Impact of HU and aging on heavy-chain CDR3s. **A**,** B** Frequencies of sequences according to CDR3 length in control vs. HU (**A**) and young vs. aged (**B**) mice. **C**,** D** Analyses of the amino acid composition of the most frequent CDR3s (11 to 14 amino acids) in control vs. HU (**C**) and young vs. aged (**D**) mice. Dispersion indexes for CDR3 amino acid composition in HU and aged mice were compared to the corresponding controls aged 3.5 months. Data are shown as the means ± SDs of 4 groups, each comprising 5 mice (*N* = 4, *n* = 5). Statistically significant differences were found using Mann–Whitney or unpaired t tests. *p ≤ 0.05; t indicates a trend

To assess the consequences of changes in IGHV and IGHD segment usage on the antigen recognition capabilities of IgM from HU and aged mice, we studied CDR3 length (Fig. 5A and B) as well as the amino acid (aa) composition of the most frequent CDR3s (Fig. 5C and D**)**. Indeed, the heavy-chain CDR3 is a major contributor to the antibody binding site because it encodes the longest and most variable loop composing this site. Figure 5A shows that HU mice did not present significant variation in CDR3 length, except for 21-aa CDR3s, which are rare. However, in aged mice (Fig. 5B), there was a significant decrease in the frequency of sequences with a CDR3 of 7, 8, 9, 11, and 18 aa and a significant increase in the frequency of sequences with a CDR3 of 14 and 20 aa. These data suggest an increase in CDR3 length with age, as previously observed by others [28]. In addition to CDR3 length, we analyzed the aa composition of the most frequent CDR3s (11 to 14 aa). Figure 5C and D show that changes in the amino acid composition of these CDR3s occurred in both HU and aged mice, suggesting that IgM binding site specificities are likely affected in these two groups of mice.

### Effects on V(D)J recombination

**Fig. 6:**
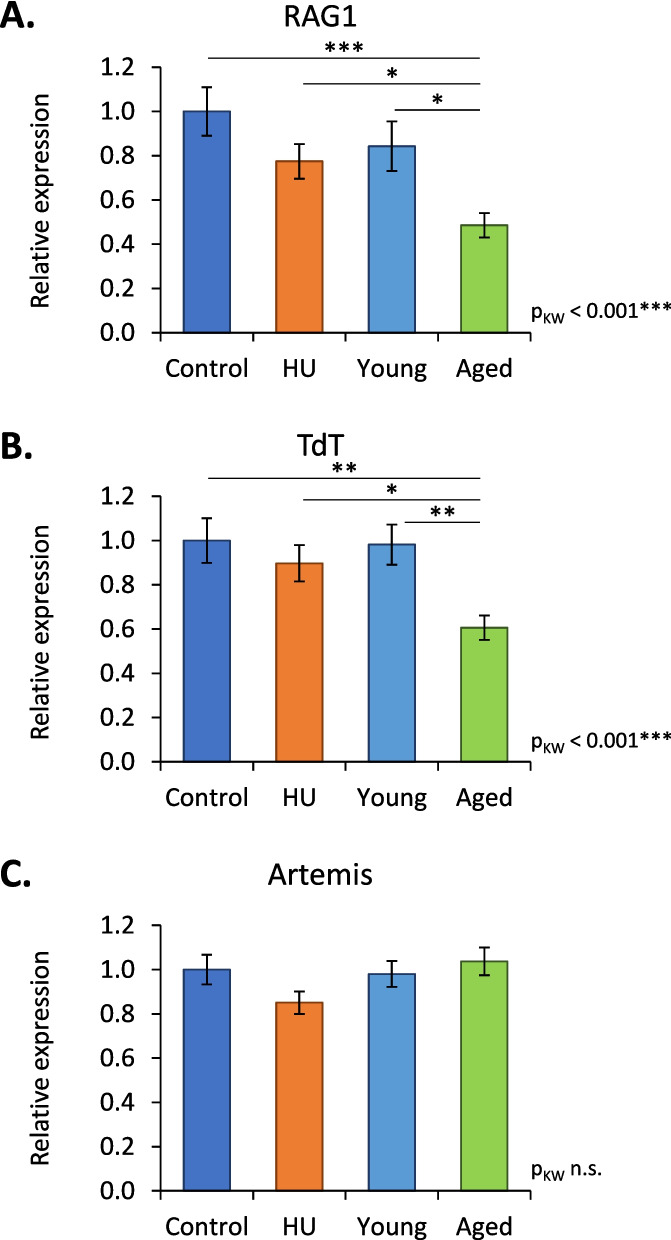
RAG1 (**A**), TdT (**B**) and Artemis (**C**) mRNA relative expression. These transcripts were analyzed by RT–qPCR in the bone marrow of HU (*n* = 24), control (*n* = 24), aged (*n* = 27) and young (*n* = 27) mice. mRNA levels were normalized to three housekeeping transcripts. The relative value obtained with controls (Control and Young groups) was set to 1. Data are shown as the means ± SDs. Statistically significant differences were found using the Kruskal–Wallis test followed by Dunn’s post hoc test. *p ≤ 0.05; **p ≤ 0.01; ***p ≤ 0.001; n.s., nonsignificant

**Fig. 7:**
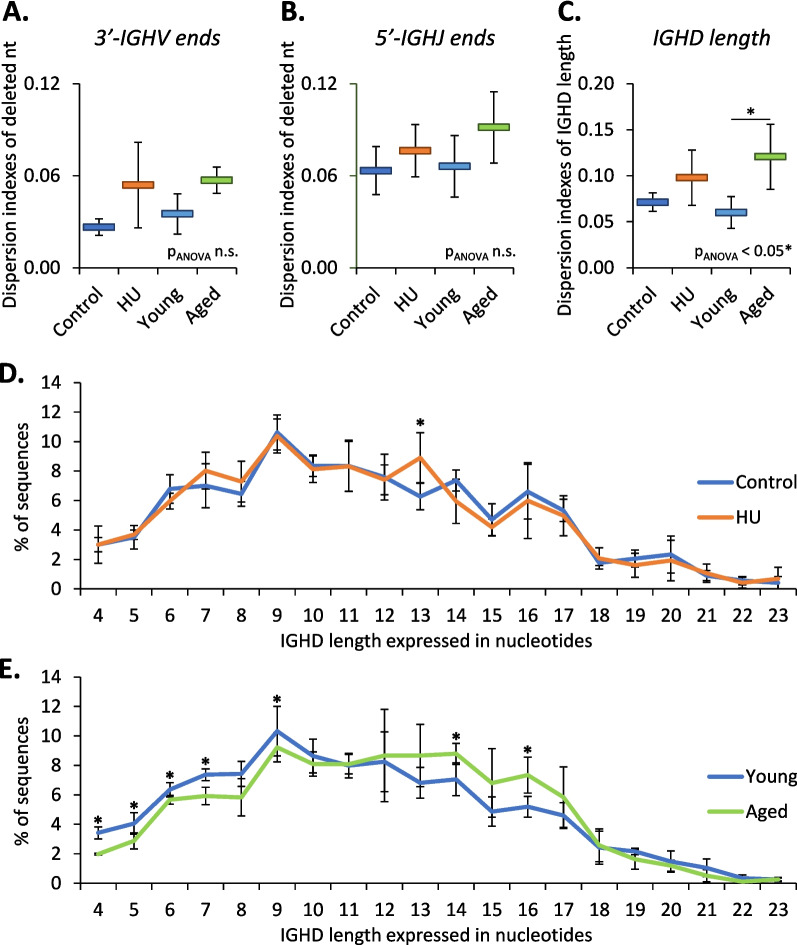
Nucleotides trimmed off IGHV, IGHJ and IGHD segments during V(D)J recombination. **A**, **B**, **C** Dispersion indexes of genomic nucleotides deleted at the 3′ end of IGHV (**A**), at the 5′ end of IGHJ (**B**), and at both ends of the IGHD segments (indicated by IGHD length) (**C**) for control vs. HU and young vs. aged mice. **D**, **E** Frequencies of sequences according to the length, in nucleotides, of IGHD segments in control vs. HU (**D**) and young vs. aged (**E**) mice. Data are shown as the means ± SDs of 4 groups, each comprising 5 mice (*N* = 4, *n* = 5). Statistically significant differences were found using one-way ANOVA followed by a Tukey–Kramer post hoc test (**A**) and Mann–Whitney or unpaired t tests (**B**). *p ≤ 0.05; n.s., nonsignificant

**Fig. 8:**
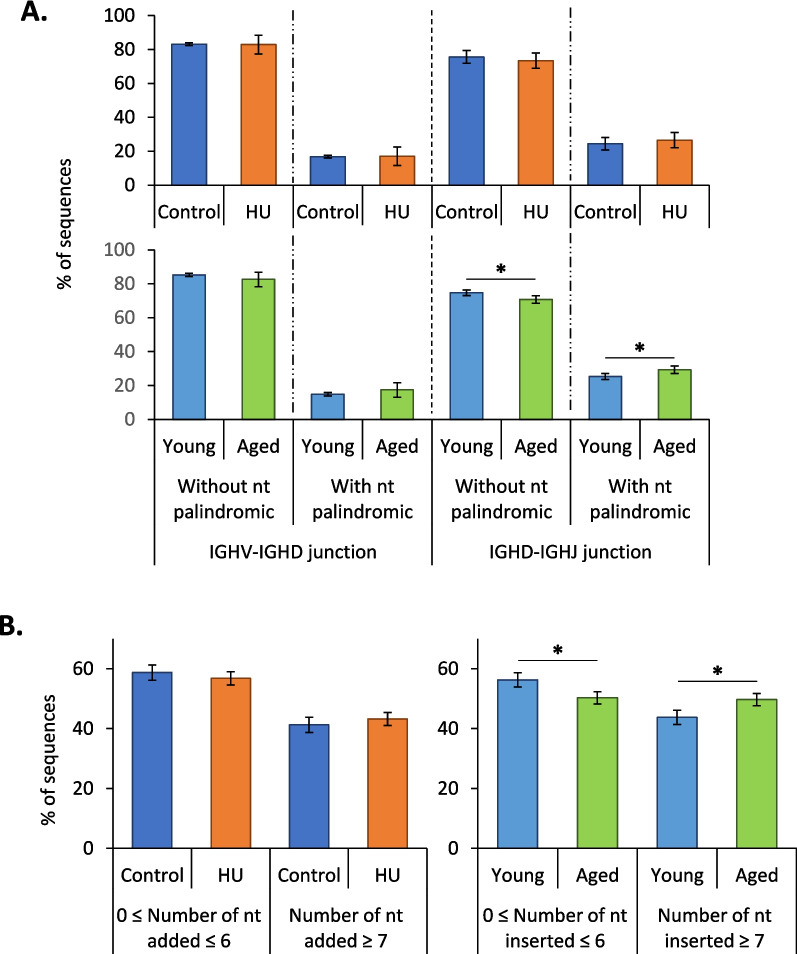
Palindromes created by Artemis and nucleotides added by TdT during V(D)J recombination. **A** Frequencies of sequences without or with palindromic nucleotides at IGHV-IGHD and IGHD-IGHJ junctions in control vs. HU and young vs. aged mice. **B** Frequencies of sequences according to the number of added nongenomic nucleotides (≤ 6 or ≥ 7 added nucleotides) in control vs. HU and young vs. aged mice. Data are shown as the means ± SDs of 4 groups, each comprising 5 mice (*N* = 4, *n* = 5). Statistically significant differences were found using Mann–Whitney or unpaired t tests. *p ≤ 0.05

To try to understand in the reasons underlying the changes in IGHV and IGHD usage, we studied the effects of HU and aging on V(D)J recombination. We first quantified RAG1, TdT and Artemis mRNAs in the bone marrow of mice from the different groups in this study. Figure 6 shows that the relative expression of transcripts encoding two of these three major effectors of V(D)J recombination, RAG1 and TdT, was significantly reduced in aged but not in HU mice. Then, we studied imprints left in IgM heavy-chain transcripts by RAG1/2, Artemis and TdT during the V(D)J recombination process. When the RAG1/2 complex cuts DNA during V(D)J recombination, some nucleotides (nt) can be deleted at the 3′ end of IGHV, on both sides of IGHD and at the 5′ end of IGHJ segments [29]. Thus, we studied the trimmed IGHV and IGHJ segments as well as IGHD length. Figure 7A, B and C show that these three signatures were not significantly affected in HU mice. However, one of them (IGHD length) was affected in aged mice. We also determined the frequencies of sequences according to the length of IGHD segments. Figure 7D shows that except in sequences containing IGHD segments of 13 nucleotides, there was no difference between the HU group and the corresponding controls. In contrast, aged mice showed a significant decrease in the number of sequences with an IGHD segment of 4 to 9 nucleotides and a significant increase in the number of sequences with an IGHD segment of 14 and 16 nucleotides (Fig. 7E). This suggests that with aging, the RAG1/2 complex removes fewer nucleotides at the ends of IGHD segments. We also analyzed palindromes created by Artemis during V(D)J recombination. The percentage of sequences without or with palindromes at IGHV-IGHD junctions was not affected by aging or HU (Fig. 8A). However, a significant increase in the frequencies of sequences with palindromes at IGHD-IGHJ junctions was observed in aged but not in HU mice, thereby indicating that the action of Artemis could be modified by aging. Finally, we examined nongenomic nucleotides added by TdT (Fig. 8B) and noted that aged mice presented an increase in the percentage of sequences with ≥7 added nucleotides, while no change was noted in HU mice. The action of TdT is therefore also likely affected by aging but not by HU. Taken together, these results indicate that the V(D)J recombination process is unlikely to be affected by 21 days of HU, while it is likely affected by aging. Thus, changes in IGHV usage observed in mice subjected to 21 days of HU (Fig. 3B) cannot be attributed to a change in the V(D)J recombination process.

## Discussion

To our knowledge, this is the first time that the bone marrow IgM repertoire of aged (18 months) C57BL/6 J mice has been studied by high-throughput sequencing. As this mouse strain is a model used in many research fields (a PubMed database search on June 05, 2023, found 25,982 results containing the word C57BL/6 J) [30], these high-throughput sequencing data deposited in the NCBI Sequence Read Archive could [31], beyond the purpose of this study, be useful to the scientific community at large.

### HU modifies the bone marrow IgM repertoire but has a weaker effect than aging

The effects of four weeks of HU on the bone marrow antibody repertoire of five 2.5-month-old unimmunized female C57BL/6 J mice were previously studied [7]. As noted here, this study reported changes in heavy-chain gene segment usage and combinations. Interestingly, modifications in the use of multiple IGHV, IGHD and IGHJ gene segments in splenic B cells and significant differences in B-cell repertoires of class-switched B cells (antigen-experienced B cells) were also reported in these mice [8]. These results combined with ours show that the antibody repertoire is affected by HU both in the bone marrow and the periphery in both sexes of the same mouse strain, although the age (2.5 vs. 3.5 months) and HU duration (4 weeks vs. 3 weeks) were not the same. In contrast, a short spaceflight (21–22 days) had no statistically significant effect on the antibody repertoire of five unimmunized 8.7-month-old female C57Bl/6Tac mice [32], while changes in the IgM repertoire of cosmonauts (median age of 48 ± 2 years) involved in long-duration space missions (ranging from 124 to 186 days) on the ISS were noted on an individual basis [4]. This suggests that the effect of spaceflight on the antibody repertoire could be conditioned by flight duration. A trend toward a lower diversity of the antibody repertoire in these C57Bl/6Tac mice that underwent spaceflight was suggested [5], as noted in aged mice [33]. Repertoire diversity is determined by two separate factors: the number of distinct B-cell lineages present and the number of different somatic mutations existing within each lineage [34]. Given our approach based on the amplification of VDJ rearrangements contained in IgM heavy chain mRNAs and the selection of unique CDR3s, the number of distinct B-cell lineages could not be determined. Indeed, a lineage or clone is defined as a set of sequences derived from the same putative VDJ rearrangement event, identified by requiring sequences to have the same IGHV segment, the same IGHJ segment, the same CDR3 length, and 90% similarity in the CDR3 [34]. Thus, we cannot make any conclusions regarding the effects of HU on IgM repertoire diversity. However, we observed increases in mean DIs for unique VDJ associations in HU and aged mice compared to their respective controls. This observation could be explained by an increase in the mutation rate in CDR3s, as in elderly individuals’ B-cell repertoires [35, 36]. Indeed, our data show that HU and aging change the amino acid composition of the most frequent CDR3s.

Our results also show that aging reduces the expression of transcripts encoding two major effectors of the V(D)J recombination process. These observations are consistent with previous works that have shown that the expression of RAG1 significantly decreases with age in human pro-B cells [37], that the percentage of pro-B cells expressing RAG2 is reduced in aged mice [38] and that there is an age-associated decline in TdT in humans and mice [39].

In addition to the effects on the bone marrow IgM repertoire, decreases in B-cell responses have previously been reported in HU [22] and aged mice [40], with a reduction in affinity maturation of antibodies, as noted when adult amphibians (*P. waltl*) were immunized during a 5-month spaceflight [12]. Furthermore, age is known to reduce the capacity to respond to vaccinations [35], and HU has been shown to delay and decrease murine antibody production in response to *Pseudomonas aeruginosa* infection [41]. Combined with the modification in the bone marrow IgM repertoire reported in the present study and our previous observation that HU leads to decreased B lymphopoiesis, similar to aging [16], these data reinforce the idea that, as suggested by Strollo and Vernikos [42], microgravity accelerates age-related processes.

Thus, alterations in the antibody repertoire combined with other immune alterations, such as persistent low-grade systemic inflammation [43–46] and changes in immune cell function [46–51], could increase the clinical impacts of spaceflight on the immune system. Indeed, it was shown that a 5-month spaceflight negatively affected amphibian antibody production in response to antigen stimulation [9–12] and that a prolonged mission on the ISS significantly modified the IgM repertoire of some cosmonauts [4]. Antigen challenge experiments will be necessary to elucidate the consequences of changes in the antibody repertoire on immunocompetence during extended space missions.

### Likely causes of IgM repertoire changes

Stress is an unlikely to cause these changes, as we did not observe increases in serum corticosterone concentration in HU and aged mice or changes in the level of NR3C1 transcripts that should decrease in response to stress [52]. Although we cannot rule out a transient increase in corticosterone in the first days of HU, this is unlikely to explain changes in the bone marrow antibody repertoire.

Age- and HU-associated bone remodeling is a highly probable contributor to changes in the antibody repertoire, as we previously demonstrated that 21 days of HU induced changes in tibial trabecular microarchitecture that were similar but less pronounced than those that occurred with age [16]. Indeed, B-cell progenitors depend on distinct bone marrow niches at different stages of differentiation [53]. Given that HU, similar to aging, leads to an imbalance between bone formation and resorption with a net loss of bone mass, it is very likely that HU, similar to aging, negatively affects endosteal niches and, consequently, committed B progenitors. As with increasing age, spaceflight is associated with osteoporosis with heterogeneity in the magnitude of bone loss among individuals [54]. It is therefore possible that bone remodeling induced by long-duration spaceflight in some individuals could contribute to explaining why the IgM repertoire of two out of five analyzed cosmonauts was significantly altered during an extended mission aboard the ISS [4]. In these two individuals, IgM repertoire changes, correlated with changes in the V(D)J recombination process and coincided with a higher stress response, suggesting that the effects on the IgM repertoire and V(D)J recombination could also depend on the development of a stress response that was not observed in our mice. As future deep-space exploration missions will be of unprecedented duration, we should consider the extent to which bone loss aggravation during such missions will affect the bone marrow antibody repertoire and consequently the immune capacities of the hosts. We should also consider whether extending HU exposure would enhance similarities with the antibody repertoire of aged mice, as it has been shown that the loss of osteoblasts is proportional to HU duration [55]. Further studies will be needed to address these important questions.

Finally, our data show that, unlike in aged mice, the changes in the bone marrow IgM repertoire of mice subjected to 21 days of HU cannot be attributed to a change in the V(D)J recombination process. Similarly, we previously showed that a model of socioenvironmental stressors encountered during spaceflight partially affects the murine TCRβ repertoire but does not affect V(D)J recombination [56]. If the V(D)J recombination process is not affected under these conditions, epigenetics appears to be another highly probable contributor to changes in the IgM repertoire. Indeed, we previously demonstrated that HU impairs IL-7 signaling in murine pro-B cells [18], which profoundly influences IGHV segment selection [20]. Additionally, we show here that HU induces changes in the use of distal IGHV segments, while age induces changes in the use of distal and median IGHV segments of the murine *IGH* locus. It has been established that the accessibility of Ig gene segments depends on epigenetic factors and chromatin conformation [19, 27, 57], and studies have reported epigenetic deregulation in response to microgravity in human blood-derived stem cells [58] and human mesenchymal stem cells [59]. A decrease in a specific histone modification, H3K27me3, was also shown at the TCRβ locus in murine thymocytes following hypergravity exposure [60]. It is also known that age affects DNA methylation and histone posttranslational modifications [61, 62]. Thus, in the future, it would be very interesting to determine the contribution of epigenetic factors to the regulation of chromatin structure in the three areas of the *IGH* locus in HU and aged mice to understand the mechanisms underlying changes in the antibody repertoire in these two situations. EZH2 methyltransferase, which is involved in the regulation of H3K27me3 levels, and H3K27me3 are interesting candidates because both are essential for the choice between proximal and distal IGHV segments [63].

### Limitations

This study is limited by the fact that we might have missed some unique rearrangements corresponding to rare B-cell clones, as indicated by a study that compared datasets generated with or without amplification [64]. However, we are confident in our results because the number of IGHV segments analyzed and detected in our libraries shows that they provide an accurate picture of IGH rearrangements. Another limitation is that bone marrow contains, in addition to naive B cells, memory and plasma B cells, the contribution of which to the modification of the IgM repertoire could not be determined since our libraries were prepared from whole bone marrow.

### Conclusion and perspectives

In conclusion, this study shows that HU, a physiological model of microgravity causing a decrease in pro-B and pre-B cells in the bone marrow [16], induced changes in the IgM repertoire within 21 days that were approximately 3-fold less than those in aged mice, which is a rapid effect. These alterations in the antibody repertoire, combined with other previously described immune alterations such as inflammation and changes in immune cell function, could increase the clinical impacts of spaceflight on the immune system, as in elderly individuals. Bone remodeling and epigenetics likely mediated the change in the IgM repertoire in HU and aged mice. Thus, countermeasures to preserve bone microarchitecture, such as physical exercise [54], omega-3 supplementation [65], or oral administration of mother-of-pearl powder [66, 67], could possibly preserve murine B lymphopoiesis and the bone marrow antibody repertoire. These examples are promising avenues for further study. It would also be interesting to compare the quality of the humoral response induced by an antigen in 2.5-month-old mice subjected to 21 days of HU and in aged mice, determine whether the effects of HU are reversible (unlike aging), and examine the effects of HU at early and late time points. Finally, it is worth noting that in addition to its relevance to spaceflight, HU is potentially a useful analog for studying the impact of physical inactivity [6], offering new insights into the contribution of sedentary behavior to immune changes.

## Methods

### Animals

C57BL/6 J male mice (2.5- and 13-month-old) were purchased from Charles River Laboratories (L’arbresles, France). Thirteen-month-old mice were kept in our animal house until they were 18 months old for comparison with 2.5-month-old mice subjected to 21 days of HU. Mice were housed in standard cages (2–3 mice per cage, 36 cm deep × 20 cm wide × 14 cm high) in a quiet room under constant conditions (22 °C, 50% relative humidity, 12-h light/dark cycles, dark period from 8 pm to 8 am) and were provided food and water ad libitum*.* Animals were treated in accordance with the French Legislation and the Council Directive of the European Communities on the Protection of Animals Used for Experimental and Other Scientific Purposes (2010/63/UE). The experiments were approved by the Lorraine Ethical Committee on Animal Experimentation (authorization CELMEA-2012-0008), and the authors complied with the ARRIVE guidelines.

### Hindlimb unloading

Two-and-a-half-month-old mice mice were isolated one week before the beginning of the experiments in HU cages manufactured according to Chapes et al. [68] (30 cm deep × 15 cm wide × 26 cm high). In the first experiment, 3 groups of 10 mice were used (control, restrained, and HU). These three groups were housed in three different vented animal cabinets (Noroit, Bouaye, France). HU mice were suspended by using a dressing retention sheet wrapped around the tail and a wire hooked on a swivel pulley system. The swivel pulley was designed to glide along two stainless steel rods that ran the length of the cage, providing a full 360° range of movement. The angle of suspension for HU mice was adjusted to 25–30° such that only the forelimbs touched the grid placed on the litter. Orthostatically restrained mice were not suspended, so all limbs were in contact with the grid. Control mice were not restrained. Throughout the 21 days of treatment, mice were provided with food and water ad libitum. Since we observed no differences in our analyses between the control and restrained groups, we decided, for the second experiment, to divide the 30 mice into two groups, 15 control mice and 15 HU mice, that were housed in two different vented animal cabinets.

### Serum and bone marrow collection

Mice were anesthetized using 5% isoflurane and then euthanized by cervical dislocation between 8 and 10 am to avoid fluctuations in corticosterone concentration due to circadian rhythm. Blood was collected by cardiac puncture, allowed to clot at room temperature for 15 min and centrifuged at 4 °C and 4000 rpm for 20 min to obtain serum samples, which were stored at - 80 °C until analysis. Bone marrow from femurs and tibias was flushed with RPMI-1640 medium. Red blood cells were lysed in 2 mL of 1x RBC lysis buffer (eBioscience, Affymetrix, Rennes, France) for 2 min on ice, and the reaction was stopped by adding 10 mL of PBS containing 2% FBS. After centrifugation at 1600 rpm and 4 °C for 5 min, the cell pellet was resuspended in 5 mL of PBS containing 2% FBS, and the cell density was determined using a Scepter™ 2.0 Cell Counter (Merck Millipore, St-Quentin-en-Yvelines, France).

### Corticosterone quantification

Corticosterone in serum samples was quantified using the DetextX® Corticosterone Enzyme Immunoassay Kit (ArborAssays, Ann Arbor, MI, USA) according to the manufacturer’s instructions. Samples were analyzed in duplicate. Absorbance at 405 nm was measured, and corticosterone concentrations, deduced from a standard curve generated using calibrators, were expressed in ng/ml.

### Flow cytometry

Bone marrow cells (5 × 10^5^) were stained with anti-B220-PE (RA3-6B2; Ozyme, Saint Quentin Yvelines, France), anti-CD43-FITC (S7; BD Biosciences, San Jose, CA, USA), anti-CD19-APC-Cy7 (1D3; Ozyme) and anti-IgM-PE-Cy7 (R6–60.2; BD Biosciences) antibodies. Anti-rat IgG2a κ isotype control FITC (RTK2758) and anti-rat IgG2a κ isotype control APC-Cy7 (RTK2758) antibodies were purchased from Ozyme. Anti-rat IgG2a κ isotype control PE-Cy7 (eBR2a) and anti-rat IgG2a κ isotype control PE (eBR2a) antibodies were purchased from eBioscience (eBioscience, Paris, France). Cells were analyzed using the Gallios flow cytometer (Beckman-Coulters, Villepinte, France) of UAR IBSLor from Lorraine University (Vandoeuvre-lès-Nancy, France). The results were analyzed using FlowJo® software (Tree Star Inc., OR, USA). Pro-B and pre-B cells were identified based on the expression of the following markers: pro-B cells (B220^lo^ CD43^+^ CD19^+^ IgM^−^) and pre-B cells (B220^lo^ CD43^−^ CD19^+^ IgM^−^).

### RT–qPCR

Total RNA was extracted from 10^7^ bone marrow nucleated cells using the NucleoSpin® RNA Plus Kit (Macherey Nagel, Hoerdt, France). RNA (1.5 μg for each sample) was reverse transcribed using random primers, RNaseOUT and M-MLV reverse transcriptase following the manufacturer’s instructions (Invitrogen, Cergy Pontoise, France). qPCRs were conducted with a Mastercycler® realplex^2^ real-time PCR machine (Eppendorf, Hamburg, Germany). Each qPCR was performed in triplicate using Takyon No ROX SYBR 2X MasterMix blue dTTP (Eurogentec, Angers, France). The cycling protocol was as follows: 3 min at 95 °C followed by 40 cycles of 15 s at 95 °C and 30 s at the annealing temperature indicated in Additional file 4. The relative expression levels of RAG1, Artemis, TdT and nuclear receptor subfamily 3 group C (NR3C1) were normalized to 3 housekeeping transcripts (elongation factor 1 alpha (Ef1a), glucuronidase-β (GUSB) and TATA-binding protein (TBP)). The stability of housekeeping transcripts was checked using Bestkeeper software [69]. Primer pairs were designed for different exons to ensure that they could not hybridize to potential traces of genomic DNA, and their specificities were checked using a Basic Local Alignment Search Tool (BLAST) search through the U.S. National Center for Biotechnology Information (Bethesda, MD, USA).

### Illumina sequencing of IGHμ transcripts

VDJ rearrangements contained in Ig μ heavy chain (IGHμ) mRNAs were amplified by 5′-RACE PCR using the SMARTer™ RACE cDNA Amplification Kit (Clontech, Palo Alto, CA, USA). For each group of mice (HU and corresponding controls, aged and corresponding controls), equal amounts (200 ng) of total RNA extracted from bone marrow nucleated cells of five mice in the same group were mixed and reverse transcribed according to the manufacturer’s instructions to reduce interindividual variability in an effort to normalize the reading. Then, two successive PCRs were performed to amplify VDJ associations. The first PCR was performed using a gene-specific primer, GSP1 (see Additional file 4), which anneals to the first constant domain of Ig μ transcripts and the UPM primer provided in the kit. The second was performed on an aliquot of the first PCR using another gene-specific primer, GSP2 (see Additional file 4), which anneals upstream of GSP1, and the NUP primer annealing to UPM. PCR products generated by this second PCR were separated on an agarose gel, and those of the expected size (0.6 kb) were purified using the Nucleospin® Gel and PCR Clean-up Kit (Macherey Nagel, Hoerdt, France). Libraries were prepared by adding Illumina adapter sequences (NEBNext® Index Primer for Illumina) to these PCR products, and high-throughput sequencing was performed from both ends (2 × 300 bp paired-end DNA sequencing) using the Illumina MiSeq sequencer of UAR IBSLor from Lorraine University (Vandoeuvre-lès-Nancy, France). Sequencing data were analyzed using IMGT/High V-quest software [70] (http://www.imgt.org) [23]. The obtained data were then analyzed using specific software (see below). Sequencing data have been deposited in the NCBI Sequence Read Archive (https://www.ncbi.nlm.nih.gov/sra) [31] under accession numbers PRJNA559699 for young/aged mice and PRJNA559675 for control/HU mice.

### Software program dedicated to the analysis of VDJ sequences

A program written in Perl was created to calculate the necessary parameters for our studies. This program parsed 16 different files, corresponding to 16 different types of samples (4 for young mice, 4 for aged mice, 4 for HU mice and 4 for control mice), produced by IMGT/HighV-quest [23]. Two to four seconds of execution was necessary to analyze a file. This execution time was proportional to the number of lines to process, with each line corresponding to 1 sequence. There were 506,399 to 960,860 lines for young mice, 332,443 to 488,167 lines for aged mice, 179,149 to 337,558 lines for HU mice, and 357,791 to 678,075 lines for control mice. The program examined each row and identified those having a CDR3 and a productive functionality. The redundancy of this set of CDR3 sequences was then calculated. A new set of results consisting of 1 representative of each CDR3 sequence expressed 1 or more times was then generated. The number of sequences with unique CDR3s ranged from 19,404 to 23,826 for young mice, 22,714 to 27,241 for aged mice, 10,406 to 20,222 for HU mice and 19,223 to 22,744 for control mice. From this set of results, the following were calculated for functional sequences: the frequency of each IGHV, IGHD and IGHJ segment; CDR3 length; the length of IGHD; the number of nucleotides deleted at 3′-IGHV and 5′-IGHJ extremities; the number of sequences having added nongenomic nucleotides; the frequencies of palindromes at IGHV-IGHD and IGHD-IGHJ junctions; and, finally, associations of all IGHV segments with all IGHJ segments for combinations having no IGHD or each IGHD segment.

### Statistical analysis

Dispersion indexes were used to determine if differences existed between control vs. HU and young vs. aged mice for the analyzed parameters. These indexes were calculated as previously described [4, 56]. GraphPad Prism 9.0 software (SAS Institute Inc., Cary, NC, USA) was used to perform statistical analyses. For two-group comparisons, the homogeneity of variance was determined using the Fisher test, and the normality of distribution was determined using the Kolmogorov–Smirnov test. When homogenous variances and distributions were observed, Student’s t tests were performed. When the variance and distribution were not homogeneous, Mann–Whitney nonparametric tests were performed. In the case of multiple comparisons, the homogeneity of variance was determined using the Bartlett test, and the normality of distribution was determined using the Shapiro–Wilk test. When homogenous variance and a normal distribution were observed, one-way ANOVA tests were performed, followed by a Tukey–Kramer post hoc test. When the variance and/or distribution were not homogeneous, Kruskal–Wallis nonparametric tests were performed followed by Dunn’s post hoc test. *P* values < 0.05 indicate significance. The results are shown as the means ± standard errors of the means (SEM).

### Supplementary Information

**Additional file 1: Table S1.** IGHV gene segments detected within our libraries of unique functional sequences.

**Additional file 2: Fig. S1.** IGHV, IGHD and IGHJ segment usage in the control groups.

**Additional file 3: Fig. S2.** Analyses of IgM CDR3s in control groups.

**Additional file 4: Table S2.** Primers used in this study.

## Data Availability

The sequencing datasets generated and analyzed during the current study have been deposited in the NCBI Sequence Read Archive (https://www.ncbi.nlm.nih.gov/sra) [31] under accession numbers PRJNA559699 for young/aged mice and PRJNA559675 for control/HU mice. All other data generated or analyzed during this study are included in this published article [and its supplementary information files].

## Abbreviations

DI: dispersion index
Ef1a: elongation factor 1 alpha
EZH2: enhancer of zeste homolog 2
GUSB: glucuronidase beta
*IGH*: immunoglobulin heavy-chain locus
IGHD: immunoglobulin heavy-chain diversity gene segment
IGHJ: immunoglobulin heavy-chain joining gene segment
IGHV: immunoglobulin heavy-chain variable gene segment
IMGT: International Immunogenetics Information System
H3K27me3: histone H3 lysine 27 trimethylation
HU: hindlimb unloading
NCBI: National Center for Biotechnology Information
NR3C1: nuclear receptor subfamily 3 group C member 1
NUP: nested universal primer
RSS: recombination signal sequence
TBP: TATA-binding protein
UPM: universal primer mix
