# Supplementary material for: Hindlimb unloading, a physiological model of microgravity, modifies the murine bone marrow IgM repertoire in a similar manner as aging but less strongly
Source: Immun Ageing. 2023 Nov 20;20:64. doi: 10.1186/s12979-023-00393-1 (PMC10659048; doi:10.1186/s12979-023-00393-1)
Supplement: Supplementary file 1 — Additional file 1: Table S1. IGHV gene segments detected within our libraries of unique functional sequences. [file 12979_2023_393_MOESM1_ESM.pdf]

Supplementary Table S1. IGHV gene segments detected within our libraries of unique functional sequences.

	IGHV subgroups																***Total***
	**VH1**	**VH2**	**VH3**	**VH4**	**VH5**	**VH6**	**VH7**	**VH8**	**VH9**	**VH10**	**VH11**	**VH12**	**VH13**	**VH14**	**VH15**	**VH16**	
**Total IGHV segments in the murine IGH locus**	111	11	9	2	24	7	4	21	4	4	2	3	2	4	2	1	211
**Functional IGHV segments according to IMGT**	67	9	7	1	12	4	3	7	4	2	2	1	1	4	1	0	125
**IGHV segments detected in our libraries of unique functional sequences**	**66**	**7**	**7**	**2**	**10**	**5**	**4**	**11**	**4**	**2**	**2**	**3**	**2**	**4**	**1**	**0**	**130**
