# Supplementary material for: Hindlimb unloading, a physiological model of microgravity, modifies the murine bone marrow IgM repertoire in a similar manner as aging but less strongly
Source: Immun Ageing. 2023 Nov 20;20:64. doi: 10.1186/s12979-023-00393-1 (PMC10659048; doi:10.1186/s12979-023-00393-1)
Supplement: Supplementary file 2 — Additional file 2: Fig. S1. IGHV, IGHD and IGHJ segment usage in the control groups. [file 12979_2023_393_MOESM2_ESM.pdf]

Figure S1

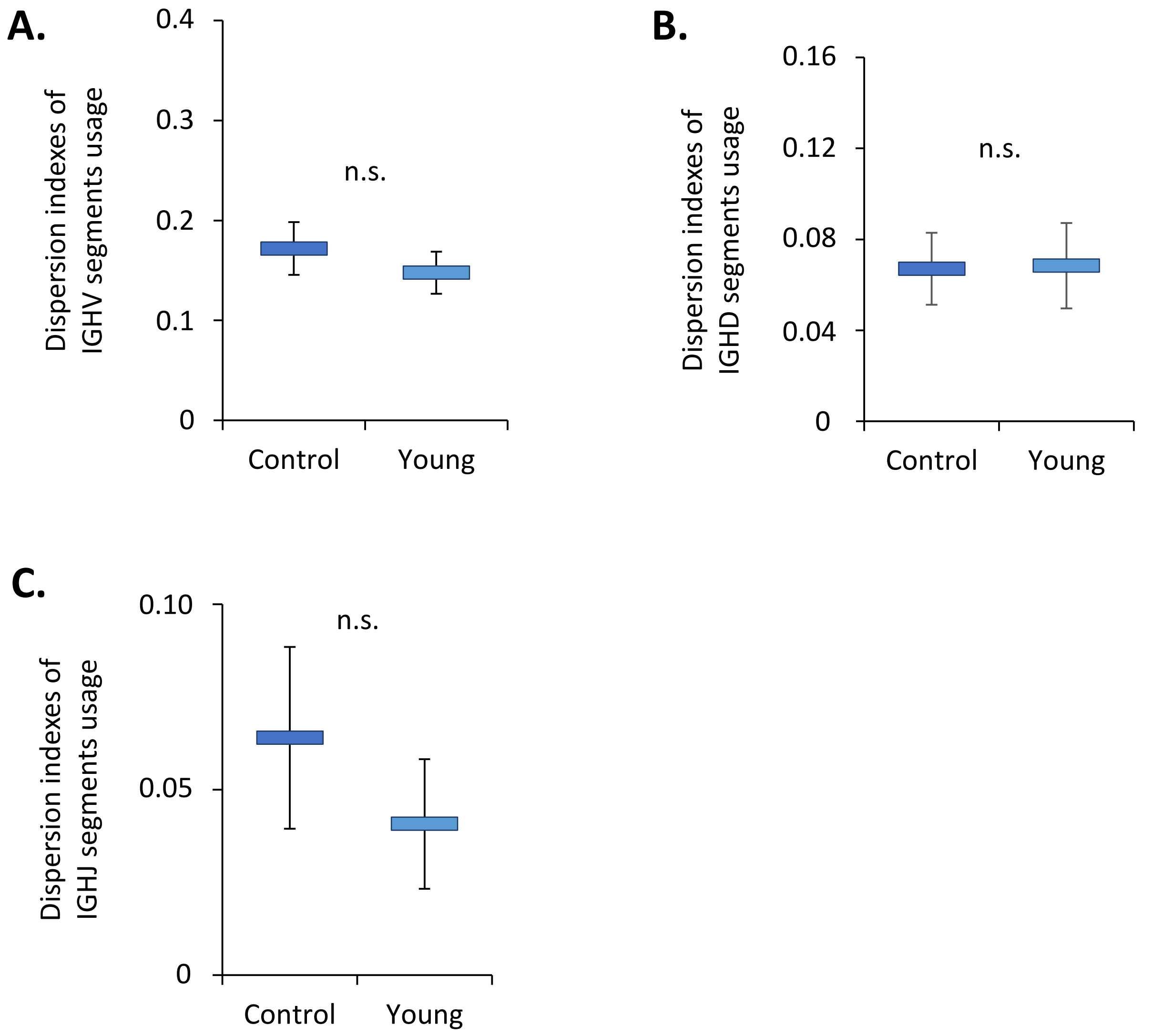

Figure S1. IGHV, IGHD and IGHJ segment usage in the control groups. (**A**) Dispersion indexes for IGHV segment usage in the bone marrow of controls of HU experiments (Control group) and of young mice of the same age as the Control group used for comparison with aged mice (Young group). (**B**) Dispersion indexes for IGHD segment usage and (**C**) dispersion indexes for IGHJ segment usage in the bone marrow of the same groups. Data are shown as the means ± SDs of 4 groups, each comprising 5 mice (N=4, n=5). No statistically significant differences were found using Mann-Whitney or unpaired t tests. n.s., nonsignificant.
