# Supplementary material for: Hindlimb unloading, a physiological model of microgravity, modifies the murine bone marrow IgM repertoire in a similar manner as aging but less strongly
Source: Immun Ageing. 2023 Nov 20;20:64. doi: 10.1186/s12979-023-00393-1 (PMC10659048; doi:10.1186/s12979-023-00393-1)
Supplement: Supplementary file 3 — Additional file 3: Fig. S2. Analyses of IgM CDR3s in control groups. [file 12979_2023_393_MOESM3_ESM.pdf]

Figure S2

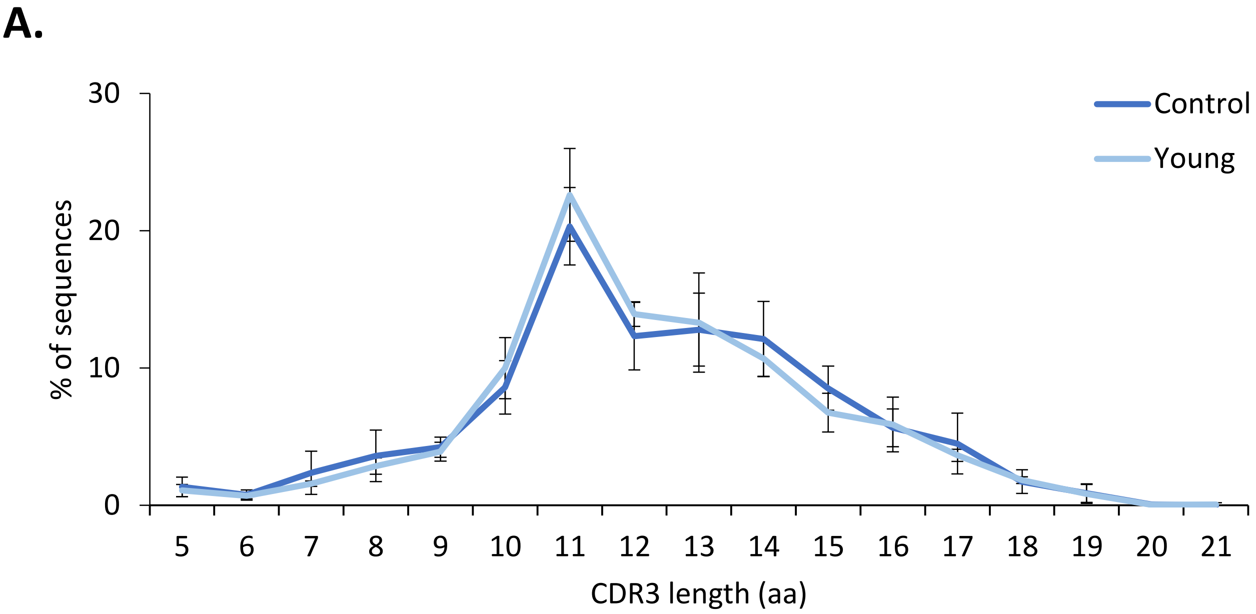

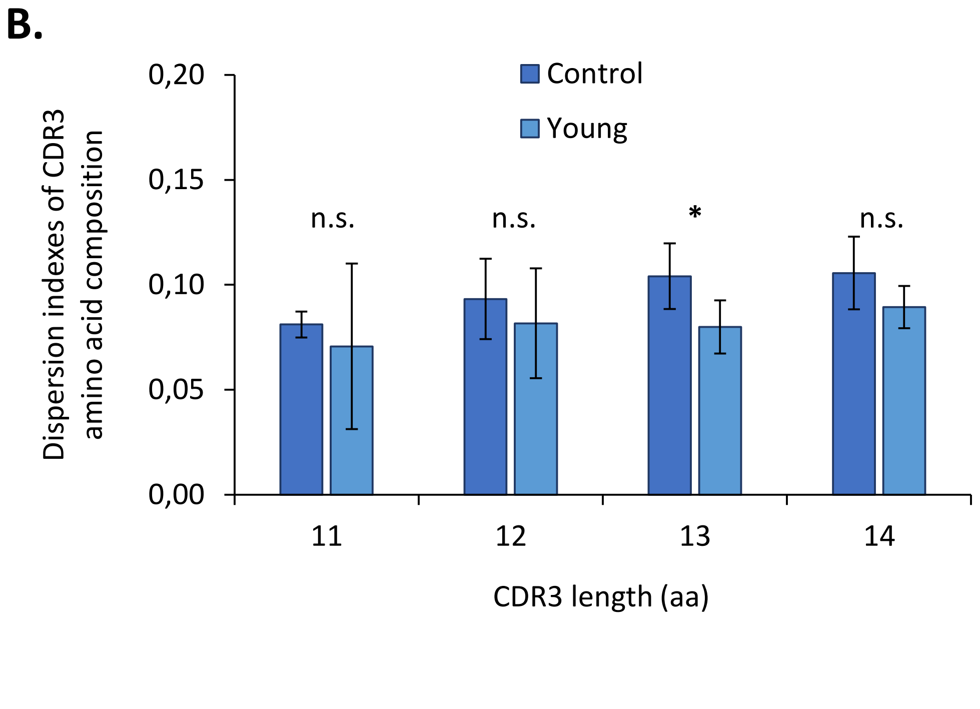

Figure S2. Analyses of IgM CDR3s in control groups. (**A**) Frequencies of sequences according to CDR3 length in controls of HU experiments (Control group) and in young mice of the same age as the Control group used for comparison with aged mice (Young group). (**B**) Analyses of CDR3s having the most frequent lengths (11 to 14 amino acids). Dispersion indexes for CDR3 amino acid composition. Data are shown as the means ± SDs of 4 groups, each comprising 5 mice (N=4, n=5). Mann-Whitney or unpaired t tests were used to analyze data. $*p \leq 0.05$. n.s., nonsignificant.
