# Supplementary material for: Hindlimb unloading, a physiological model of microgravity, modifies the murine bone marrow IgM repertoire in a similar manner as aging but less strongly
Source: Immun Ageing. 2023 Nov 20;20:64. doi: 10.1186/s12979-023-00393-1 (PMC10659048; doi:10.1186/s12979-023-00393-1)
Supplement: Supplementary file 4 — Additional file 4: Table S2. Primers used in this study. [file 12979_2023_393_MOESM4_ESM.pdf]

Supplementary Table S2. Primers used in this study. F, forward; R, reverse.

	Sequences	PCR product length (bp)	Annealing temperature (°C)
	**qPCR**		
Ef1a	F 5'-AGAACCAGGCCAGAACCGAA-3'<br>R 5'-GCAGCTGAGACTCCTTTCCA-3'	183	60.8
GUSB	F 5′-CCGATTATCCAGAGCGAGT-3′<br>R 5′-CTCAGCGGTGACTGGTTCG-3′	197	60.8
TBP	F 5′-CAAACCCAGAATTGTTCT-3′<br>R 5′-ATGTGGTCTTCCTGAATCCCT-3′	131	58
RAG1	F 5′-GGCTCAGTCTACATCTGTACA-3′<br>R 5′-ACGGACTCATGATACGGATTG-3′	143	59.2
TdT	F 5′-CGAAGACCTCGTTAGCTGTG-3′<br>R 5′-TCATGTCCAGTCATCTTACCC-3′	140	60.8
Artemis	F 5′-CTAGGAGTCCGCACCACGT-3′<br>R 5′-GGTGCAGGATATCAGGCATG-3′	147	60
NR3C1	F 5'-CAAGGGTCTGGAGAGGACAA-3'<br>R 5'-TACAGCTTCCACACGTCAGC-3'	220	60.8
	**5′RACE**		
GSP1<br>UPM<br>GSP2<br>NUP	5'-GAAGGAAATGGTGCTGGGCAGGAAGTCC-3′<br>5'-CTAATACGATCACTATAGGGCAAGCAGTGGTATCAACGCAGAGT-3'<br>5'-TACACGACGCTCTTCCGATCTAGACAGGGGGCTCTCGCAGGAGACGA-3'<br>5'-AGACGTGTGCTCTTCCGATCTAAGCAGTGGTATCAACGCAGAGT-3'	600-650	68
